# Seasonal differences in the effects of local concentrations of atmospheric substances and meteorological elements on asthma exacerbation of children in metropolitan area, Korea: A 13-year retrospective single-center study

**DOI:** 10.3389/fped.2023.1028901

**Published:** 2023-04-28

**Authors:** Jae Kyoon Hwang, Jae Yoon Na, Kyung Suk Lee, Jae-Won Oh, Young-Jin Choi

**Affiliations:** ^1^ Department of Pediatrics, Hanyang University Guri Hospital, Guri, Republic of Korea; ^2^ Department of Pediatrics, Hanyang University Seoul Hospital, Seoul, Republic of Korea; ^3^ Department of Pediatrics, Hanyang University College of Medicine, Seoul, Republic of Korea

**Keywords:** air pollutants, asthma exacerbation, meteorological element, mold, pollen

## Abstract

**Purpose:**

Air pollutants contribute to asthma exacerbation, and the types of air pollutants involved in acute asthma exacerbation may differ depending on climate and environmental conditions. This study aimed to identify factors affecting asthma exacerbation in each of the four seasons so that to prevent acute asthma exacerbation and to establish effective treatment strategies for each season.

**Methods:**

Pediatric patients aged 0–18 years old hospitalized or admitted to the emergency room for asthma exacerbation at Hanyang University Guri Hospital between January 1, 2007, and December 31, 2019 were recruited. The number of asthma exacerbations comprised the total number of patients admitted to the emergency room or hospitalized for asthma and treated with systemic steroids. The association between the number of asthma exacerbations/week and average concentrations of atmospheric substances and meteorological elements in that week were analyzed. Multiple linear regression analyses were performed to examine the association between various atmospheric variables and the number of asthma exacerbations.

**Results:**

The number of asthma exacerbations was found to be associated with the concentration of particulate matter with an aerodynamic diameter of ≤10 μm in that week in autumn. No atmospheric variables exhibited an association in other seasons.

**Conclusions:**

Air pollutants and meteorological factors affecting asthma exacerbation vary by season. Moreover, their effects may change *via* their interaction with each other. The results of this study suggest that it will be helpful to establish differentiated measures for each season to prevent asthma exacerbation.

## 1. Introduction

The World Health Organization defines air pollution as contamination of the indoor or outdoor environment by any chemical, physical, or biological agent that adversely modifies the natural characteristics of the atmosphere (1). These agents directly affect human health (2, 3). Metropolitan areas are characterized by high population densities and high levels of transportation and industrial activities that jointly deteriorate the atmospheric environment (4, 5). Moreover, most residential areas and schools are located near roads, which can frequently present a threat of exposure to air pollutants such as particulate matter (PM), ozone (O_3_), carbon monoxide (CO), nitrogen dioxide (NO_2_), and sulfur dioxide (SO_2_) (4–6). Recently, there has been a growing interest in PM, which has been reported to adversely affect patients with asthma by increasing their hospitalization rate, worsening respiratory symptoms, and hindering lung function (3, 7, 8). Pollen is a seasonal aeroallergen that causes allergies; accordingly, increased pollen concentration cause asthma exacerbation (9, 10).

Asthma is the most common chronic respiratory disease in childhood globally (11). Despite specific guidelines for the treatment of childhood asthma and improvements in asthma management, acute asthma exacerbations continue to occur, placing a considerable burden on both medical finances and pediatric patients and their families (12). Therefore, prevention of exacerbation is an important part of the treatment goal for asthma. Acute asthma exacerbation is defined as rapid worsening of asthma symptoms, such as coughing, shortness of breath, wheezing, and chest tightness (11–13).

PM with an aerodynamic diameter of ≤10 μm (PM_10_), SO_2_, NO_2_, O_3_, and CO plays a major role in asthma occurrence and exacerbation, as well as the course of this disease (14, 15). Other factors such as humidity, temperature, and temperature differences, all of which depend on seasonal variations, also contribute to asthma exacerbations (13). Weather changes increase emergency department visits and hospitalizations among patients with asthma (16). Extremely high temperatures are associated with increased emergency room visits by patients with asthma (17, 18). A previous study conducted in the United States found that summer heat significantly increased hospitalization rate for asthma, as well as that a decrease in winter temperature and an increase in SO_2_ concentration were significantly associated with acute asthma exacerbation (19).

Types of air pollutants and meteorological elements involved in acute asthma exacerbation could be differ depending on climate and environmental conditions. Therefore, in the present study, we divided the year in Korea into four seasons and explored the factors that may be manipulated to prevent asthma exacerbations and establish seasonal-effective treatment approaches. The results of this study will contribute to the development of seasonal strategies for coping with exacerbations in patients with asthma.

## 2. Materials and methods

### 2.1. Ethical considerations

This study was approved by the Institutional Review Board (IRB) of the Hanyang University Guri Hospital, Gyeonggi-do, South Korea (IRB No. 2022-07-029). The requirement for written informed consent was waived owing to the retrospective nature of the study. This study was conducted in accordance with the principles of the Declaration of Helsinki.

### 2.2. Participant recruitment

Concentration of air pollutants greatly varies across regions. Therefore, we limited our study to a suburban area of Seoul (Gurisi, Gyeonggi-do, South Korea), which provides good access to the Hanyang University Guri Hospital for nearby residents. Gurisi is a city with 188,550 residents occupying an area of 33.31 km^2^ (population density: 5,670.25/km^2^). The city has a small mountain with a green index of 43 and a relatively small area of 211,699 m^2^. There are no industrial complexes or large highways nearby, and most of the city's territory is taken by residential buildings that are 20–30 years old.

To analyze the association between asthma exacerbation and atmospheric substances to which the patients were locally exposed, only patients with no major difference between their place of residence and the main place of stay, such as school and workplace, were included in this study. Since most adults spend considerable time working, the separation between their home and working areas should be taken into account, as concentrations of atmospheric substances and pollen (in the external environment) of their residence areas may not represent their true exposure. In Korea, children attend kindergartens and schools in close vicinity from their home address. Therefore, most children living in Gurisi attend kindergartens and schools in the same area, so there is no difference between their place of residence and the place where they spend most of their daytime. Accordingly, we recruited only the patients aged <19 years old living in the designated study areas. Children aged 0–18 years old admitted to the emergency room or hospitalized for asthma at Hanyang University Guri Hospital between January 1, 2007, and December 31, 2019 were recruited. The patients were recruited only until the end of 2019 because, with the outbreak for COVID-19 pandemic, the exposure to air pollution has decreased due to reduced vehicle traffic and outdoor activity, which could interfere with the study analysis (20, 21).

### 2.3. Definition of asthma exacerbation

Asthma exacerbation was defined as admittance to the emergency room or hospitalization for asthma and treated with systemic steroids.

### 2.4. Exclusion

Respiratory diseases such as respiratory viral infections cause asthma exacerbation, constituting a variable that should be evaluated. However, considering that viral confirmatory tests are not performed in the emergency room, it was difficult to confirm whether the patients had respiratory viral infections. Therefore, we excluded patients with respiratory infectious diseases, such as bronchiolitis, bronchitis, or pneumonia, in addition to asthma.

Furthermore, we also excluded the patients who did not reside in Gurisi after confirming their residence through a chart review.

### 2.5. Air pollutants

PM and air pollutant concentrations were measured in Gurisi. Daily and weekly average concentration data for PM_10_, PM_2.5_, O_3_, NO_2_, CO, and SO_2_ were collected from January 1, 2007, to December 31, 2019 using the data published by the Ministry of Environment (www.airkorea.or.kr).

There was one center in Guri, Gyeonggi-do, to measure air quality and two centers to measure air pollutants in the region. The distance between the two measuring stations was 2.3 km.

Among these centers, the data measured at a station near the hospital were used. Given that pollen and mold are being measured in the hospital, we attempted to measure the concentration of atmospheric substances in the same area. The concentration of SO_2_ was measured using the pulsed UV-fluorescence method. CO concentration was measured with the non-dispersive infrared method and nitrogen dioxide (NO_2_ concentration) concentration using the chemiluminescence method. The concentration of fine dust (PM-10) was assessed using the x-ray absorption method, while that of ozone (O_3_ concentration) was evaluated using the UV photometric method.

### 2.6. Pollen and mold

Pollen was collected at Hanyang University Guri Hospital from January 1, 2007, to December 31, 2019. Pollen distribution was measured daily by installing a 7-day recording volumetric spore trap (Burkard Manufacturing Co., Hertfordshire, UK) at the height of 1.5 m from the surface of the hospital roof. We collected weekly drums that collected pollen from the air, and these were further examined by two specialists. The glycerin-adhesive vinyl was stained with Calberla fuchsin solution (10 ml of glycerin, 20 ml of 95% alcohol, 30 ml of distilled water, and 0.2 ml of basic fuchsin) and identified under an optical microscope with 400-fold magnification (OLYMPUS/BX43, Tokyo, Japan). The number of pollen grains/species/m^3^ was calculated and recorded. Pollen was categorized according to its size, shape, and surface pattern depending on the allergy-related plants distributed in each region.

The number of mold/spores/m^3^ was also recorded and calculated. The mold was categorized according to its size and shape.

### 2.7. Meteorological elements

The meteorological elements were measured in Gurisi. Daily and weekly average temperature, humidity, and precipitation data were collected from January 1, 2007, to December 31, 2019 using the data published by the Meteorological Agency (www.weather.go.kr).

### 2.8. Statistical analyses

In order to reduce the possible effect of variation in atmospheric environmental variables, such as temperature and humidity, we performed our analysis within a period of similar environmental variables. Therefore, correlations between atmospheric factors and asthma exacerbation were analyzed per each of the four seasons (spring, summer, autumn, and winter). Spring was defined from March to May, summer from June to August, autumn from September to November, and winter from December to February. For each individual week, we analyzed the association between the weekly number of asthma exacerbations and the average weekly concentration of air pollutants, pollen, and meteorological elements. First, cross-correlation coefficients between weekly measures of each environmental substance and asthma exacerbation were computed to determine which time point (t, t-1, t-2, or t-3) had the most influence. The time point abbreviations were as follows: (t) for that week, (t-1) for 1 week prior, (t-2) for 2 weeks prior, and (t-3) for 3 weeks prior. The associations between atmospheric factors that showed correlation during that period and the number of asthma exacerbations/week were investigated by multiple linear regression analysis to identify the factors with the greatest influence. Statistical significance in all analyses was set at *p*-value < 0.05, and all analyses were performed using SAS version 9.4 (SAS Inc., Cary, NC, USA).

## 3. Results

### 3.1. Demographic characteristics

A total of 633 patients (mean age, 6.58 ± 4.19 years; *n* = 391 males) experienced asthma exacerbations in the past 13 years.

The numbers of exacerbations in spring, summer, autumn, and winter in the data were 168 (26.5%; mean age, 7.32 ± 3.11 years; *n* = 92 males), 157 (24.8%; mean age, 4.98 ± 2.65 years; *n* = 85 males), 292 (46.1%; mean age, 6.79 ± 5.27 years; *n* = 102 males), and 131 (20.7%; mean age, 7.24 ± 2.96 years; *n* = 79 males), respectively. The number of exacerbations was the highest in autumn.

### 3.2. Spring

In spring, the average levels of O_3_, CO, temperature, and humidity in a week (t) had the greatest correlation with the number of asthma exacerbations in that week (t). Moreover, the average concentration of PM10 1 week prior (t-1) also strongly correlated with the number of asthma exacerbations in a given week (t) (Table 1). The concentrations of other substances showed no correlation. The results of computing the association between the number of asthma exacerbations at (t)/week and the average concentrations of atmospheric substances at (t)/week using multiple linear regression revealed no correlation between asthma exacerbations and substance concentrations in that week (t) and 1 week prior (t-1).

**Table 1:** Cross-correlation coefficients between the number of asthma exacerbations (t) and atmospheric substance (t-n) in spring.

In spring	t-3	t-2	t-1	t
PM_10_ ***ρXY*** (***h***)	0.018	0.069	**0** **.** **110**	0.070
O_3_ ***ρXY*** (***h***)	0.021	0.063	−0.011	**0**.**124**
CO ***ρXY*** (***h***)	−0.109	−0.091	−0.035	**0**.**111**
Temperature ***ρXY*** (***h***)	0.064	.0031	0.104	**0**.**143**
Humidity ***ρXY*** (***h***)	−0.089	−0.017	0.042	**0**.**119**

Bold values represent highest cross-correlation coefficients.

t-n: n weeks ago.

***ρXY*** (***h***): cross-correlation coefficient.

O_3_, ozone; CO, carbon monoxide.

### 3.3. Summer

In summer, concentrations of PM_10_, O_3_, and tree pollen during that week affected asthma exacerbation. The concentrations of other substances showed no correlation (Table 2). The results of multiple regression analysis with the concentrations and the number of asthma exacerbations in that week revealed no correlation between the number of asthma exacerbations and substance concentrations during that week (t).

**Table 2:** Cross-correlation coefficients between the number of asthma exacerbations (t) and atmospheric substance (t-n) in summer.

In summer	t-3	t-2	t-1	t
PM_10_ ***ρXY*** (***h***)	0.002	−0.037	−0.053	**0** **.** **122**
O_3_ ***ρXY*** (***h***)	0.055	−0.042	−0.068	**0**.**194**
Tree pollen ***ρXY*** (***h***)	0.024	−0.132	0.046	**0**.**159**

Bold values represent highest cross-correlation coefficients.

t-n: n weeks ago.

***ρXY*** (***h***): cross-correlation coefficient.

PM_10_, particulate matter 10; O_3_, ozone.

### 3.4. Autumn

In autumn, concentrations of most environmental substances (PM_10_, O_3_, weed pollen, mold) in that week correlated with the number of asthma exacerbations in a given week (t). Concentrations of other substances showed no correlation (Table 3). The results of computing the association between the number of asthma exacerbations at (t)/week and the average concentrations of atmospheric substances that showed correlation at (t)/week using multiple linear regression revealed that the number of asthma exacerbations was associated with PM_10_ during that week (adj. R^2^ = 0.273) (Table 4).

**Table 3:** Cross-correlation coefficients between the number of asthma exacerbations (t) and atmospheric substance (t-n) in autumn.

In autumn	t-3	t-2	t-1	t
PM_10_ ***ρXY*** (***h***)	0.030	0.007	0.057	**0** **.** **137**
O_3_ ***ρXY*** (***h***)	0.055	−0.110	0.044	**0**.**164**
Weed pollen ***ρXY*** (***h***)	0.005	0.010	0.039	**0**.**129**
Mold ***ρXY*** (***h***)	0.046	0.086	0.055	**0**.**177**

Bold values represent highest cross-correlation coefficients.

t-n: n weeks ago.

***ρXY*** (***h***): cross-correlation coefficient.

PM_10_, particulate matter 10; O_3_, ozone.

**Table 4:** A multiple linear regression analysis of the number of asthma exacerbations (t)/week (y) and correlated atmospheric substance (t) in autumn.

	Estimate $\left(b_{i}\right)$	Std. error	*p*-value
PM10	**0**.**0182**	**0**.**002013**	**<0**.**001**
O_3_	21.2514	18.532169	0.1341
Weed pollen	3.5299	11.378256	0.8290
Mold	697.1223	881.575954	0.4639

Bold values represent highest cross-correlation coefficients.

PM_10_, particulate matter 10; O_3_, ozone.

The number of asthma exacerbation (t) = *b*0 + *b*1 × PM10(t) + *b*2 × O_3_ (t − 1) + b3 × Weed pollen (t) + b4 × Mold (t).

### 3.5. Winter

In winter, the average levels of O_3_, humidity, and weed pollen in that week (t) exhibited the greatest correlation with the number of asthma exacerbations in a given week (t). Moreover, average concentrations of NO_2_ and CO at 1 week prior (t-1) showed the greatest correlation with the number of asthma exacerbations in a given week (t). Concentrations of other substances showed no correlation (Table 5). The results of computing the association between the number of asthma exacerbations at (t)/week and the average concentrations of atmospheric substances at (t)/week using multiple linear regression revealed that the number of asthma exacerbations showed no association with substance concentrations in that week (t) and 1 week prior (t-1).

**Table 5:** Cross-correlation coefficients between the number of asthma exacerbations (t) and atmospheric substance (t-n) in winter.

In winter	t-3	t-2	t-1	t
O_3_ ***ρXY*** (***h***)	0.009	0.085	−0.065	**0** **.** **131**
NO_2_ ***ρXY*** (***h***)	0.055	0.068	**0**.**163**	0.058
CO ***ρXY*** (***h***)	0.010	0.015	**0**.**103**	0.009
Humidity ***ρXY*** (***h***)	−0.022	−0.019	0.066	**−0**.**126**
Weed pollen XY (***h***)	0.006	0.045	0.027	**0**.**121**
Mold XY (***h***)	0.064	0.016	0.001	**0**.**186**

Bold values represent highest cross-correlation coefficients.

t-n: n weeks ago.

***ρXY*** (***h***): cross-correlation coefficient.

O_3_, ozone; NO_2_, nitrogen dioxide; CO, carbon monoxide.

## 4. Discussion

We found that, in most seasons, asthma exacerbations were greatly influenced by the mean concentrations of environmental substances in that week and the previous week; however, the impact of the substances differed by season. Accordingly, for each season, we ran multiple linear regression analyses to identify the association between asthma exacerbations and environmental substance concentrations in that week and 1 week prior. In autumn, PM_10_ concentrations in that week (t) were associated with asthma exacerbation at time (t). In spring, summer, and winter, no substances were associated with asthma exacerbation at both time (t) and time (t-1).

Our finding on the varying impact of atmospheric substances on asthma exacerbation suggests that different seasons have different actions and require distinctive precautions to prevent asthma exacerbations. Unlike in the other three seasons, in autumn, there was a correlation between the increased PM10 concentration and asthma exacerbation. Therefore, in the autumn season, patients with asthma should carefully check the air pollution forecast system and respond accordingly.

The number of asthma exacerbations patients in autumn was the highest (*n* = 292) across all four seasons. The larger the number of asthma exacerbations, the more correlated the analysis results would be. In future research, further well-characterized, longitudinal, and large-scale cohort studies would be needed to clarify seasonal differences in asthma exacerbation. Future research should also establish an appropriate countermeasure against asthma exacerbations for each season.

Air pollution worsens asthma symptoms, and there is robust evidence showing its associations with increased airway hyperresponsiveness, use of symptom relievers, and emergency room visits and hospitalizations (22, 23). According to the results of previous time series studies, air pollution in urban areas is also associated with an increase in the number of deaths from respiratory diseases (24–26). In Korea, pollen and air pollutant concentrations vary largely by region, with a significant difference between Seoul and Busan (10, 27). While several recent studies on asthma exacerbation have been conducted on significant amounts of data (28), analyses using local concentrations remain scarce, highlighting the need for research the correlation between local exposure and asthma exacerbation. Contrary to existing investigations involving big data, such as on a national scale, in this study, we examined the concentrations of atmospheric substances as close to the site of exposure as possible (28). Therefore, this was a single-center study conducted at a secondary hospital with good accessibility, and only the patients living in a certain area were examined.

In our results, we found differences in factors affecting each season, and the time point was also different. Air pollution aggravates the symptoms in patients with asthma, increasing emergency room visits and hospitalizations. Weather factors, such as temperature and humidity, adversely affect the course of asthma, and factors contributing to the exacerbation of asthma symptoms interact with each other to produce different effects. Therefore, there is a difference in the timing and degree of influence by season. In the present study, when each factor was analyzed individually, the concentration of atmospheric substances in that week or the previous week for each season exhibited a strong correlation with the number of asthma exacerbations. However, when all substances were analyzed together by multiple regression analysis, no association between the number of asthma exacerbations and atmospheric substances was noted in most seasons, except for in autumn.

This study has several limitations. First, the study was conducted on children, and the results should not be generalized to adults. To analyze the association between the number of asthma exacerbations and atmospheric substances to which the patients were exposed locally, only the patients with no significant difference between their residence and their main living radius were included in the study. Most adults spend considerable time working, and their exposures in living and working areas can considerably vary. Accordingly, the concentrations of atmospheric substances and pollen in adult patients' residences may not represent exposure. Therefore, we recruited only the patients aged <19 years old living in the designated study areas. In the future, we will conduct a larger cohort study to recruit adults with no difference in the radius of action and analyze the correlation between local exposure and asthma exacerbation in adults. Second, respiratory diseases such as respiratory viral infections cause asthma exacerbation and can contribute to asthma exacerbation. However, since viral confirmatory tests are not performed in the emergency room, it was difficult to confirm whether the patients had respiratory viral infections. However, effort was made to exclude exacerbation conditions due to viral infections to a significant extent by excluding patients with respiratory infectious diseases, such as bronchiolitis, bronchitis, or pneumonia, in addition to asthma at the time of admission to the emergency room and/or hospitalization. We intend to supplement the present results with further larger-scale research that would involve multiple centers, broader time, and patient scope.Third, we did not factor in the variable of indoor air pollutant concentrations. To date, several previous studies showed that indoor air quality can be worse than outdoor air quality (29). However, indoor air quality improved with the development of ventilation systems (29, 30). Therefore, it would also be useful to define air pollutant concentrations with respect to the level to which the patients are locally exposed indoors. Fourth, in order to clarify the local influence of atmospheric substances, we designated a characteristic small area called Gurisi and recruited only the patients from that area. Gurisi is a metropolitan area characterized by a low green area ratio and abundance of residential buildings. Several studies suggest that increased green space reduces allergy occurrence and exacerbation (31). So, our results could be biased. Finally, there were more environmental variables that interacted with each other than just those analyzed in this study. The amount of green space also affects asthma exacerbation (31). To reduce the effect of differences in atmospheric environmental variables, we attempted as to perform our analysis strictly within a period when such factors were similar. Therefore, correlations among air pollution, pollen concentration, and asthma exacerbation were analyzed by studying spring, summer, autumn, and winter separately. We also recruited only those patients who lived in a certain state area in a similar environment with no significant difference in land conditions, such as the amount of green space.

Despite these limitations, our results revealed seasonal differences in the correlation between asthma exacerbation and local concentrations of environmental substances, including allergic pollen and meteorological elements.

In conclusion, air pollution and meteorological factors interact with each other in asthma exacerbation. Air pollution causes worsening of symptoms in patients with asthma, and meteorological factors, such as temperature and humidity, can also affect the course of the disease. However, their effects may change through interaction with air pollutants (32, 33). Accordingly, our results show that air pollutants and meteorological factors that affect asthma exacerbation vary by season. In autumn, controlling PM10 concentrations was important to prevent asthma exacerbation. No significant correlation was found in other seasons; however, further well-characterized, large-scale cohort studies are expected to demonstrate seasonal differences in the effects of local concentrations of atmospheric factors on asthma exacerbation in children.

## Data Availability

The original contributions presented in the study are included in the article/supplementary material, further inquiries can be directed to the corresponding author.
